# Low Fouling Nanostructured Cellulose Membranes for Ultrafiltration in Wastewater Treatment

**DOI:** 10.3390/membranes13020147

**Published:** 2023-01-23

**Authors:** Ritika Joshi, Nilay Sebat, Kai Chi, Madani Khan, Ken I. Johnson, Abdulrahman G. Alhamzani, M. A. Habib, Tom Lindstrom, Benjamin S. Hsiao

**Affiliations:** 1 Department of Chemistry, Stony Brook University, 100 Nicolls Road, Stony Brook, NY 11794, USA; 2 Department of Chemistry, Imam Mohammad Ibn Saud Islamic University (IMSIU), Riyadh 11623, Saudi Arabia; 3 KTH Royal Institute of Technology, 100 44 Stockholm, Sweden

**Keywords:** cellulose membranes, nanocellulose, ultrafiltration, low fouling, wastewater treatment

## Abstract

Ultrafiltration (UF) is a common technique used in wastewater treatments. However, the issue of membrane fouling in UF can greatly hinder the effectiveness of the treatments. This study demonstrated a low-fouling composite cellulose membrane system based on microfibrillated cellulose (MFC) and silica nanoparticle additives. The incorporation of ‘non-spherical’ silica nanoparticles was found to exhibit better structural integration in the membrane (i.e., minimal aggregation of silica nanoparticles in the membrane scaffold) as compared to spherical silica. The resulting composite membranes were tested for UF using local wastewater, where the best-performing membrane exhibited higher permeation flux than commercial polyvinylidene difluoride (PVDF) and polyether sulfone (PES) membranes while maintaining a high separation efficiency (~99.6%) and good flux recovery ratio (>90%). The analysis of the fouling behavior using different models suggested that the processes of cake layer formation and pore-constriction were probably two dominant fouling mechanisms, likely due to the presence of humic substances in wastewater. The demonstrated cellulose composite membrane system showed low-fouling and high restoration capability by a simple hydraulic cleaning method due to the super hydrophilic nature of the cellulose scaffold containing silica nanoparticles.

## 1. Introduction

The sustainable management of water resources and efficient treatment of wastewater are essential to tackle the challenge of water scarcity, especially in developing countries. Ultrafiltration (UF) is a commonly used separation technique in wastewater treatment and an efficient tool to separate a wide range of contaminants. It is a low-pressure membrane filtration process, in which source/contaminated water is passed through a semi-permeable membrane under a trans-membrane pressure that can separate suspended solids, bacteria, viruses, etc [1]. UF membranes are typically defined by their pore sizes in the range of 10 nm–100 nm. It is a well-understood separation technology, having been through continuous development over decades, which finds its applications across various industries such as dairy production, chemical recovery, medical use, cell harvesting, and water treatment. UF is can be an essential process in wastewater treatment because of its ability to effectively remove turbidity, organic (humic substances) matters, etc [2]. The existing UF membranes are derived from synthetic polymers, such as polyvinylidene fluoride (PVDF), polyacrylonitrile (PAN), polysulfone (PS), and polyethersulfone (PES) [3]. These membranes have good mechanical strength and chemical and thermal stability. However, these synthetic polymeric membranes are prone to deterioration in permeability due to the accumulation of solids, suspended particles, etc. on the membrane surface and/or within the pores [4,5]. The deposition of suspended particles, macromolecules, etc. on the membrane surface and inside the pore walls can lead to membrane fouling, reducing the permeate flux temporarily or permanently. Membrane fouling would result in increased operational costs due to the increases in labor, energy consumption, maintenance, etc. and limits the broader application of other UF processes, such as drinking water production [6].

**Figure 1:**
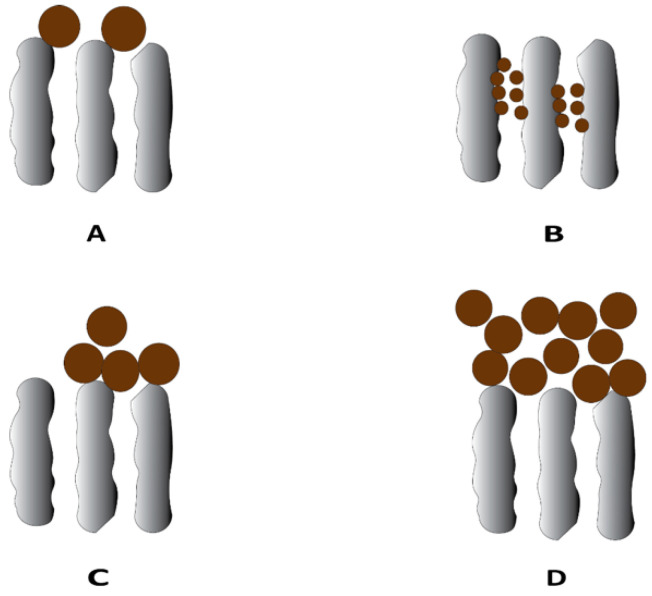
Typical fouling mechanism in the ultrafiltration process: (**A**) complete blocking, (**B**) standard blocking, (**C**) intermediate blocking, and (**D**) cake layer formation.

Membrane fouling can be categorized into two types, depending on their relative resistance to the membrane cleaning process—reversible and irreversible fouling [7]. Reversible fouling refers to fouling that can be removed by cleaning or backwashing treatments. Irreversible fouling is fouling that remains even after cleaning. Additionally, fouling can be hydraulically irreversible or chemically irreversible. Membrane fouling in UF membranes can propagate through different mechanisms depending on the interaction between the membrane surface and foulants, including adsorption, pore blocking, and cake/gel layer formation [8] (Figure 1). Adsorption occurs due to specific interacting forces between the foulants and membrane surface in the form of van der Waals forces, electrostatic attractions, or chemical bonding and is a common mechanism in the case of having proteins and humic acids as foulants. Pore blocking is the deposition of foulant particles leading to full or partial closure of pores, whereas cake layer formation is the layer-by-layer deposition of foulants on the external membrane surface, which can lead to an additional resistance to the permeation flux. Cake layer formation usually occurs when suspended solids, small colloidal particles, and macromolecules are present as foulants [8].

Fouling in the UF process can be affected by several factors: membrane structure properties, material characteristics, and operating conditions. Modifications of the membrane surface are frequently used to improve the fouling-resistance of the membrane. For example, introduction of a hydrophilic layer/surface on the membrane can improve its interaction with water molecules and useful to reduce the membrane fouling tendency [9]. In other words, the formation of a hydration layer can the membrane surface can hinder the interaction of foulants with membrane surface and enable the accumulated fouling layer to be easily removed. There have been many efforts to increase the hydrophilicity of the polymeric membrane materials by methods such as surface coating [9], blending [10], and plasma treatment [11]. Typically, these methods involve complex chemical treatments on the synthetic polymer membranes (typically hydrophobic). Additionally, synthetic membrane polymers can be combined with varying hydrophilic nanofillers (e.g., SiO_2_, TiO_2_, ZnO, and graphene oxide (GO)) to create hydrophilic surface using techniques such as non-solvent induced phase separation (NIPS) [12] or thermally induced phase separation (TIPS) [13]. However, one major challenge of these methods is the agglomeration of nanofillers in the polymer scaffold [14].

Cellulose nanofibers are promising scaffolding materials for UF membranes due to their low dimensionality (and thus, low pore size), good sustainability, large functional surface, good cost-effectiveness, and low environmental impact [15]. Moreover, cellulose nanofibers exhibit high hydrophilicity due to the presence of abundant hydroxyl and carboxyl groups on their surface. There have been several studies on the application of cellulose nanofibers as a barrier layer in UF membranes. Previous works in our group demonstrated the use of (2,2,6,6-Tetramethylpiperidinyloxyl) TEMPO-oxidized cellulose nanofibers as a barrier layer on electrospun PAN/PET substrate to fabricate high flux and low fouling hierarchical ultrafiltration membranes for water purification [16,17,18]. These studies on thin-film nanocomposite membranes (TFNC) confirmed the role of oxidized cellulose nanofibers as a low-fouling material due to their highly hydrophilic and charged nature [19]. However, these studies involved electrospun polymer materials such as PAN and PET (polyethylene terephthalate) as the substrates to fabricate low-fouling UF membranes.

In this study, we demonstrated a superhydrophilic low-fouling membrane using nanostructured carboxymethylated cellulose fibers as scaffold materials and silica nanoparticles as hydrophilic additives through a simple fabrication method. Colloidal silica nanoparticles are commonly used as reinforcing agents for fibrous materials [20]. These inorganic particles are biocompatible, inexpensive, and abundant. The addition of negatively charged silica nanoparticles could further tune the pore size as well as antifouling properties of the membranes. There have been some studies on employing spherical silica nanoparticles in membrane fabrication by chemical techniques, such as grafting and blending [21]; however, spherical silica nanoparticles tend to aggregate in the membrane scaffold. We hypothesize that the ‘non-spherical’ or chain-like structure of silica nanoparticles can reduce the tendency of filler aggregation, and hence, create a more homogeneously consolidated structure in the nanostructured cellulose fibers. To test this hypothesis, we compared the incorporation of both non-spherical and spherical silica nanoparticles and also compared their structural integration with cellulose fibers and their effects on the UF performance in wastewater treatment. Positively charged polyamide amine epichlorohydrin (PAE) was used to strengthen the wet-integrity of cellulose fibers as well as help the retention of negatively charged silica nanoparticles [22]. The resulting composite membranes were characterized in terms of structure, morphology, and hydrophilicity, and their UF performance was evaluated by local wastewater and compared with commercial polymeric membranes. The antifouling performance was further analyzed using different theoretical models to investigate the likely fouling mechanism during wastewater UF.

## 2. Experimental

### 2.1. Materials

Never-dried (totally chlorine free) TCF-bleached sulfite dissolving pulp (trade name: Dissolving Plus) from a mixture of Norway spruce (60%) and Scottish pine (40%) was obtained from Domsjö Fabriker, Örnsköldsvik, Sweden. All chemicals in the carboxymethylation reaction were ACS grade reagents and were used without further purification. Specifically, monochloroacetic acid, sodium bicarbonate, ethanol, isopropanol, sodium hydroxide, hydrochloric acid, and acetic acid were purchased from Fisher Scientific, Rochester, NY, USA. De-ionized (DI) water was used in all reactions unless otherwise specified. Polyamideamine epichlorohydrin (Kymene 920A) was obtained from Solenis, Wilmington, DE, USA. The spherical silica nanoparticles (Ludox TM-50) were purchased from Sigma Aldrich, St Louis, Missouri, USA in the form of a 50 wt% aqueous suspension. The ‘non’ spherical silica nanoparticles (Levasil RD442) in the form of a 15 wt% aqueous suspension were provided by Nouryon, Stenungsund, Sweden. Three commercial grade ultrafiltration (UF) membranes–PVDF A6 (MWCO 500 kDa), PVDF V6 (MWCO 500 kDa), and PES LX (MWCO 300 kDa) were purchased from the Sterlitech Corporation, Auburn, WA, USA.

### 2.2. Preparation of Carboxymethylated Microfibers

Wood pulp fibers were chemically pretreated before the carboxymethylation reaction. In specific, pulp fibers (30 g) were dispersed in de-ionized water overnight at a stirring speed of 350 rpm. The dispersed fibers were then solvent exchanged with ethanol (300 mL) using the intermediate filtration approach. The fibers were then dispersed in a solution, consisting of 3 g of monochloroacetic acid in 150 mL of isopropanol, for 1 h. Separately, a solution containing 4.5 g of NaOH, 145 mL of methanol, and 550 mL of isopropanol was prepared at 60 °C in a 3 L round bottom flask. The pulp fibers dispersion was added to the heated reaction solution in the round bottom flask to complete the carboxymethylation reaction for 1 h under reflux conditions.

After the carboxymethylation reaction, resulting fibers were washed in three steps—first with de-ionized water, second with 600 mL of 0.1 M acetic acid, and finally with de-ionized water again until the conductivity was below 5 µS/cm. Carboxymethylated fibers were subsequently dispersed in 600 mL of 4 wt% sodium bicarbonate solution at room temperature for 1 h to convert the carboxyl groups into their sodium ionic form. The resulting fibers were then washed with de-ionized water again until the conductivity became lower than 5 µS/cm. These carboxymethylated fibers were homogenized with a high-pressure homogenizer (Panda Plus, 2000) to partially defibrillate the fibers. In this study, fiber dispersions with concentrations between 0.5–0.9 wt% were homogenized at different pressure conditions (100–350 bar) to obtain microfibrillated celluloses (MFC) with different degree of delamination (fibrillation).

### 2.3. Membrane Fabrication

**Figure 2:**
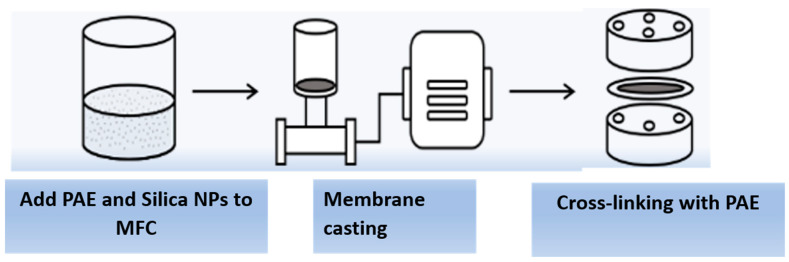
Schematic of the membrane fabrication process for the cellulose membrane.

The cellulose membrane was prepared from MFC or partially defibrillated cellulose fibers. A schematic illustration of the membrane fabrication procedure is shown in Figure 2. In this procedure, polyamideamine epichlorohydrin (PAE) and silica nanoparticles (NPs) were added to the MFC dispersion with a weight ratio from 0.5 to 0.8 wt%. Specifically, PAE was first added to the MFC (0.250 g) aqueous dispersion at a rate of 1 mL/min followed by the addition of silica NPs at a rate of 1 mL/min. The mixture was then rigorously stirred with a magnetic stirrer at 2000 rpm and room temperature for 30 min. The membrane fabrication was carried out by vacuum filtration using a 0.65 µm DVPP filter (Millipore) to obtain the uniform basis weight (i.e., 50 g/m^2^). After filtration, the membranes were mounted in special drying frames and heated at 110 °C for 10 min to initiate the cross-linking reaction between the azetidinium group in PAE and the carboxyl group in MFC. The resulting membrane was conditioned for at least 18 h at room temperature before any further testing or characterization.

### 2.4. Sample Characterization

#### 2.4.1. Charge Determination of Carboxymethylated Microfibers

The carboxylate content of carboxymethylated microfibers (prior to homogenization) was determined by conductometric titrations. In this study, dry carboxymethylated microfibers (~2 g) were dispersed in deionized water for 30 min, where the pH value was raised to 2 by adding 0.01 M HCl. The fibers were washed several times to remove excess HCl until the conductivity was below 5 µS/cm. This procedure removed any unwanted metal contaminants from the fibers, which were then dispersed in 0.001 M NaHCO_3_ for 30 min at pH = 9 (adjusted by the addition of 0.1 M of NaOH). The recovered fibers were then washed again with deionized water until the conductivity became lower than 5 µS/cm. This procedure removed some likely contaminants such as adsorbed hemicellulose from the fibers. The total charge density of carboxymethylated microfibers was determined by conductometric titrations of the fibers prior to homogenization. The washed fibers were soaked in 0.1 M HCl for 45 min twice and washed with deionized water until constant conductance was obtained. The fibers were then dispersed in 300 mL of 0.001 M NaCl and titrated against 0.1 M NaOH. The conductivity of the dispersion was monitored by a conductivity meter (Oakton, COND6+ Series).

#### 2.4.2. Fourier Transform Infrared Spectroscopy

MFC, silica NPs, and hybrid cellulose membranes were characterized by Fourier infrared spectroscopy (Thermo Scientific, Nicolet iS10). The spectra were obtained using the attenuated total reflectance (ATR) mode in the wavenumber range of 500–4000 cm^−1^ and at a resolution of 4 cm^−1^.

#### 2.4.3. Electron Microscopy Measurements

The morphological analysis of MFC and hybrid cellulose membranes was done by scanning electron microscopy (SEM, Zeiss, LEO 1550 SFEG) equipped with the setup of energy dispersive spectroscopy (EDS). In the sample preparation for SEM, membrane specimens were taped on a stainless-steel plate for the image acquisition. The transmission electron microscopy (TEM) measurement was carried out on a JEOL JEM 1400 instrument operated at an accelerating voltage of 120 kV equipped with a GATAN ORIUS CCD camera. In the sample preparation for TEM, a 10 µL aliquot of MFC suspension (0.01 wt%) or silica NP suspension (0.01 wt%) was deposited on the carbon-coated Cu grid (300 mesh, Ted Pella Inc., Redding, CA, USA). To increase the electron density contrast of cellulose, the MFC samples were stained with 2.0 wt% uranyl acetate before air drying.

#### 2.4.4. Dynamic Light Scattering (DLS) and Zeta Potential Measurements

DLS measurements to determine the size and distribution of silica NPs were performed on a NanoBrook Omni Particle Sizer instrument (Brookhaven Instrument, Holtsville, NY, USA) using a 40 mW red diode laser. Three repeat measurements were made at 25 °C. Zeta potential measurements were carried out using the NanoBrook Omni instrument (Brookhaven Instrument Inc. Holtsville, NY, USA), where five repeat measurements were also made at 25 °C.

#### 2.4.5. Porosity and Pore Size Measurements

Porosity measurements of the cellulose membranes were conducted by the gravimetric method. In this study, the membrane samples were first dried for 5 h in an oven and then weighed by an analytical balance. The dried samples were then submerged in isopropyl alcohol for 1 h in capped vials and weighed again after the removal of the excess solution. The porosity of the membrane was calculated using the following equation:

(1) $$\varepsilon =\frac{V_{pore}}{V_{Total}}=\frac{\frac{m_{IPA}}{\rho_{IPA}}}{\frac{m_{IPA}}{\rho_{IPA}}+\frac{m_{polymer}}{\rho_{polymer}}}$$

where *V* is the volume, *m_IPA_* and *ρ_IPA_* are mass and density of isopropyl alcohol, respectively, and *m_polymer_* and *ρ_polymer_* are the mass and density of the membrane, respectively. Three independent measurements were made, where the average value was reported.

The pore size determination was done by the solute-rejection method [23] using polystyrene beads of different diameters (i.e., 0.5, 0.3, 0.2, 0.1, and 0.05 µm) as the model compounds. The rejection ratio of the specific polystyrene bead by the membranes was calculated by determining the concentration of the filtrate using UV-vis spectrophotometry (ThermoFisher Genesys^TM^ 10S).

#### 2.4.6. Contact Angle and Tensile Strength Measurement

The hydrophilicity of the cellulose and commercial membranes was measured by a contact angle goniometer (OCA 15EC, Dataphysics Instruments, Charlotte, NC, USA) using the sessile drop method. In this measurement, a 4 µL droplet of de-ionized water was deposited on the membrane sample taped on a glass slide, where the water contact angle on the membrane surface was determined using the video image captured by the built-in software.

The tensile strength of MFC membranes was measured by cutting MFC membrane was cut into small strips of 1.5 mm to 6.5 mm, weighed, and measured in stress-strain mode.

### 2.5. Performance Evaluation for Wastewater Ultrafiltration

The performance of the cellulose membrane was evaluated against municipal wastewater using a dead-end filtration system (Model HP4750X, Sterlitech Corporation, Auburn, WA, USA). The wastewater sample was obtained from a local wastewater treatment plant (Stony Brook Sewage Treatment Plant, Stony Brook, NY, USA) after being treated in a bioreactor and before further purification. The wastewater samples were stored in the refrigerator at 5 °C and replaced every 14 days. The total suspended solids (TSS) and total dissolved solids (TDS) concentration of wastewater were 2690 ± 634.5 mg/L and 551 ± 102.4 mg/L, respectively. The membranes, with an effective surface area of 14.6 cm^2^, were compressed with de-ionized water for 60 min at a pressure of 0.5 bar before filtration experiments. The permeate flux (*J*, L m^−2^ h^−1^) for wastewater was calculated using the following equation:

(2) $$J=\frac{V}{A\times t}$$

where *V* is the permeate volume, *A* is the membrane area, and *t* is the filtration time. The separation efficiency of the membrane was measured in terms of turbidity, TDS, and TSS concentrations of the feed (C_o_) and the permeate (C_t_), respectively. The turbidity was measured using a turbidity meter (Thermo Scientific Orion AQ3010).

### 2.6. Fouling Study

The fouling behavior of the cellulose and commercial membranes was analyzed by dynamic ultrafiltration experiments. In the fouling study, filtration was conducted with the wastewater sample for 2 h at a feed pressure of 0.5 bar using the same dead-end filtration system mentioned above, where the permeate flux (*J*) was measured. At the end of the 2 h UF study, the membranes were taken out of the cell for analysis as follows. The fouled membranes were first cleaned with de-ionized water for 1 min without permeation. The de-ionized water was then filtered through the membrane at 0.5 bar for 1 h, where the water flux (*J*_1_, L m^−2^ h^−1^) post-cleaning was measured. The antifouling performance of the membrane was determined in terms of flux recovery ratio (FRR), calculated by the following equation [14]:

(3) $$\mathrm{FRR}\;\left(\%\right)=\frac{J_{1}}{J_{o}}\times 100$$

where *J*_1_ is the permeate flux and *J_o_* is the initial flux. The flux loss by reversible fouling ratio (*R_r_*) and irreversible fouling ratio (*R_ir_*) was calculated by the following equations:

(4) $$R_{r}=\frac{J_{1}-J}{J_{O}}\times 100$$

(5) $$R_{ir\;}=\frac{J_{O}-J_{1}}{J_{O}}\times 100$$

### 2.7. Modeling of the Membrane Fouling Mechanism

The flux decline due to the membrane fouling in a dead-cell ultrafiltration study can be explained by different fouling mechanisms—complete blocking, standard blocking, intermediate blocking, and cake filtration. The equations [24,25] for different membrane fouling mechanisms are given in Table 1. Theoretical modeling for these fouling mechanisms was performed to identify the likely fouling mechanism for the hybrid cellulose membrane (MFC-NP) challenged with wastewater treatment.

## 3. Results and Discussion

### 3.1. Structure and Property of MFC or Partially Fibrillated Carboxymethylated Cellulose Fibers

**Figure 3:**
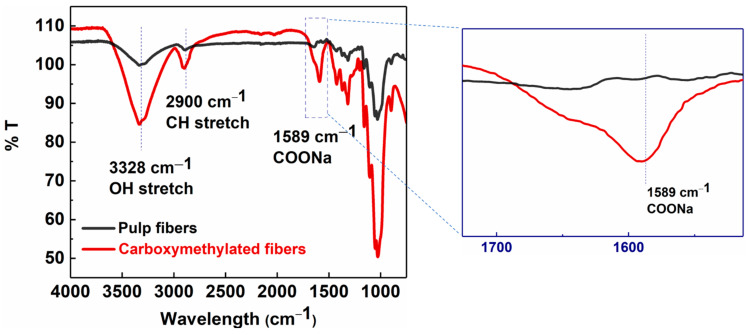
FTIR analysis of carboxymethylated (red) and pulp fibers (black).

Wood pulp fibers were pre-treated and functionalized via the carboxymethylation procedure and then partially defibrillated using high-pressure homogenization to obtain MFC. The conversion of surface hydroxyl groups on pulp fibers to carboxymethyl groups on carboxymethylated cellulose fibers was confirmed by the FTIR analysis (Figure 3). The FTIR results showed the appearance of a strong adsorption band at 1589 cm^−1^, representing the C=O stretching vibrations of the carboxylate group, on carboxymethylated cellulose fibers after the pretreatment [26].

In Figure 3, the IR spectra of pulp fibers and carboxymethylated cellulose fibers (carboxy fibers) showed typical cellulose peaks at 3328 cm^−1^ for the O-H stretching, 2900 cm^−1^ for the CH_2_ stretching, 1310 cm^−1^ for the CH_2_ rocking, 1160 cm^−1^ for the C-O-C stretching, and 1030 cm^−1^ for the CH ring vibrations [27]. The carboxylate content of carboxymethylated fibers calculated by the conductometric titration method was equal to 0.410 mmol/g. The mechanical energy in the form of high-pressure homogenization was used to partially defibrillate the chemically pretreated fibers into MFC. Homogenization was carried out at different fiber concentrations (0.5–0.9 wt%) at varying pressures (100–350 bar) to achieve dispersions of the different fiber sizes and distributions.

A gravimetric method was employed to qualitatively estimate the apparent degree of delamination in these dispersions [28]. In this analysis, the dispersions were diluted to 0.03% (*w*/*w*) and stirred overnight using a magnetic stirrer at 1000 rpm. The diluted samples were centrifuged at 1000× *g* for 15 min, to separate larger fiber fragments. The concentrations before (*c_bc_*) and after (*c_ac_*) the centrifugation were used to estimate the fraction of the ‘nano-sized’ cellulosic materials or degree of delamination (*c_N_* (*w*/*w*)%) in the dry content of the dispersion using the following equation:

(6) $$c_{N}\;\left(\frac{w}{w}\right)\%=\frac{c_{ac}}{c_{bc}}\times 100$$

where *c_N_* denotes the fraction of entities that can resist phase separation during centrifugation. This method assumes that the value of *c_N_* increases with the increasing efficiency of the delamination process. A comparison of the apparent degree of delamination at different conditions of homogenization and estimated energy consumption [29] is given in Table 2.

**Figure 4:**
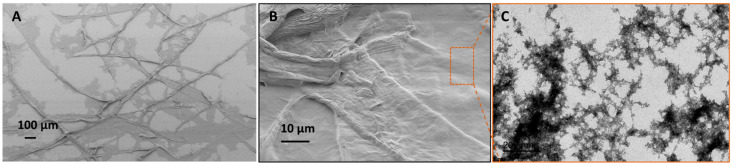
(**A**,**B**) SEM image of partially defibrillated carboxymethylated fibers after homogenization, (**C**) TEM image of nano-scale fibers separated by centrifugation.

In Table 2, it was seen that the fiber dispersion obtained by homogenization of 0.9% (*w*/*w*) for 1 pass at 100 bar exhibited a broader fiber size distribution (e.g., an average fiber width in the range of 20–25 µm, and fiber length in several hundred microns, as shown in the SEM image of Figure 4A,B). The nano-scale fragments in the dispersion could be separated by centrifugation of the 0.03% (*w*/*w*) fiber dispersion at 1000× *g* for 15 min. TEM analysis of these nano-scale fibers was conducted to analyze the morphology of nanofibers. The TEM image in Figure 4C shows long entangled fibers of several hundred microns in length with a width in the range of 10–20 nm on average. These MFC obtained by partial defibrillation of carboxymethylated cellulose fibers were used as a scaffold to fabricate porous UF membranes, as described earlier.

### 3.2. Nanostructured Cellulose-Silica Membranes

**Figure 5:**
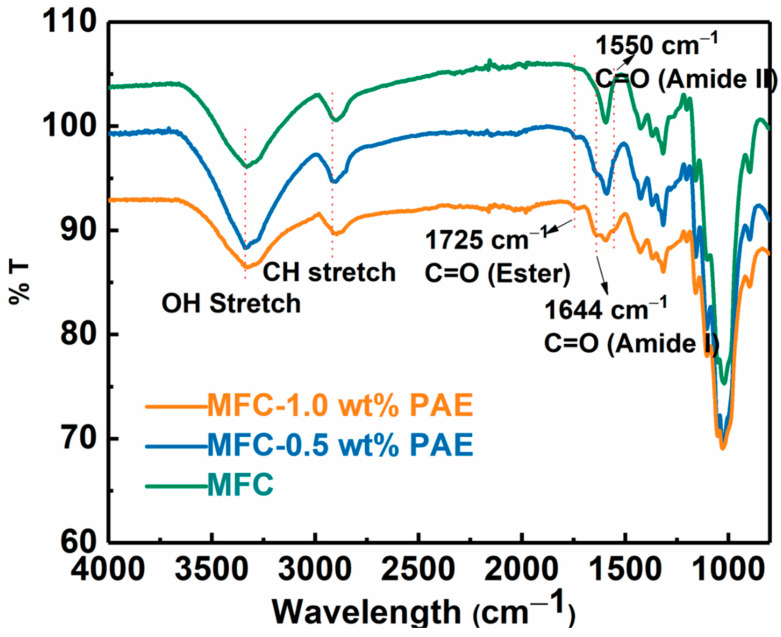
FTIR analysis of MFC and PAE cross-linked MFC membranes (with different PAE concentrations).

The wet-strength of the MFC scaffold was enhanced by cross-linking reaction using polyamideamine-epichlorohydrin (PAE) through wet-end addition prior to membrane casting followed by heat treatment. After cross-linking, the negatively charged carboxylate groups on MFC can form a covalent ester bond with positively charged azetidinium group of PAE molecules [30]. These new covalent linkages strengthen the inter-fibrillar bonding between cellulose fibers in the wet environment, and hence, greatly improve the wet integrity of the fibrous network [31,32]. The formation of the ester bond was confirmed by the FTIR analysis of the PAE cross-linked MFC membranes as shown in Figure 5. In this figure, the peak around 1725 cm^−1^ could be identified, which confirmed the formation of the ester bond (i.e., C=O stretching) between the carboxylate group on MFC and azetidinium groups of PAE [33]. The spectra also showed vibrational peaks around 1640 cm^−1^ (Amide I C=O) and 1550 cm^−1^ (Amide II N-H), further verifying the presence of PAE in the membranes. There was also a slight increase in intensities of these new peaks due to the increase in PAE concentration with cross-linking.

**Figure 6:**
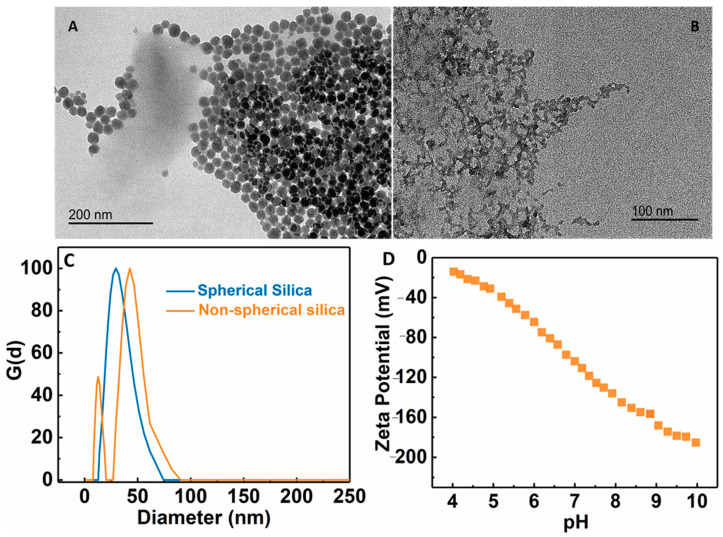
TEM images of (**A**) spherical silica NPs, (**B**) non-spherical silica NPs, (**C**) dynamic light scattering analysis of spherical and non-spherical silica NPs, and (**D**) zeta potential of non-spherical silica NPs as a function of the pH value.

Pore size is a key parameter in the UF membrane. Depending on the application, its pore size is usually in the range of 10 nm–0.1 µm. To control the pore size of the membrane, the PAE cross-linked MFC membrane was further modified by the addition of silica NPs. To accomplish this goal, different types of negatively charged silica NPs (wet-end additives or nanofillers) were added to adjust the pore size of the membrane. Two silica NPs: spherically shaped (S) and ‘non’ spherical or chain-like silica (NS) were explored for this purpose. Dynamic light scattering (DLS) analysis of spherical NPs showed a single peak at around 23 nm, while there were two peaks for non-spherical silica centered around 10 nm and 50 nm (Figure 6C). It is clear that the non-spherical NPs exhibited a non-homogeneous distribution of structure [34]. TEM images of these silica NPs are shown in Figure 6A,B, exhibiting corresponding spherical and chain-like structures, as indicated by DLC. Figure 6D showed the zeta potential scan of the non-spherical silica NPs as a function of the pH value, indicating that the surface charge on the particles remained negatively charged over the entire pH range.

**Figure 7:**
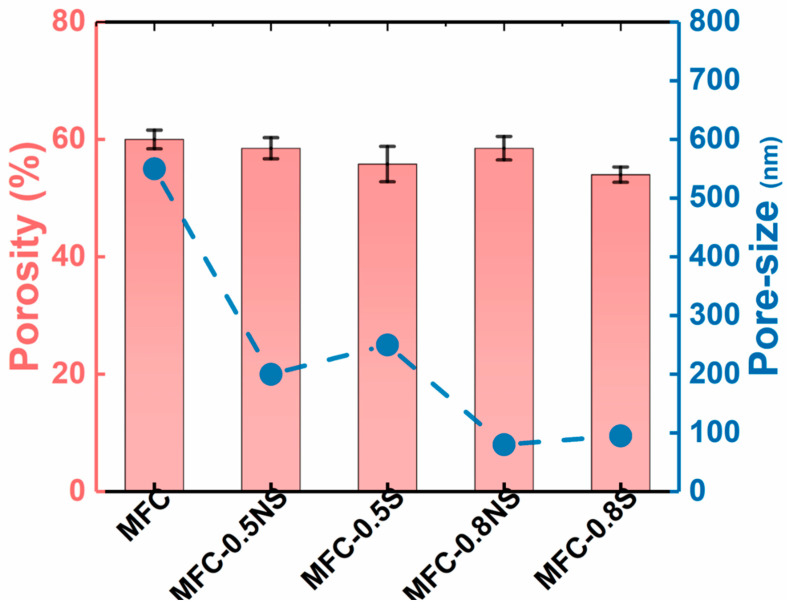
Porosity and pore size determination of MFC-NS and MFC-S membranes with varying silica NP concentrations.

The pore size of MFC membrane typically depends on the size distribution of delaminated fibers and the thickness of the membrane. The addition of silica NPs, both spherical and non-spherical, helped reduce the average pore size of the membranes from 0.5 µm for pure MFC membrane to below 0.1 µm for MFC-0.8S (i.e., the MFC dispersion was added with a weight ratio of 0.8 wt% spherical NPs) and MFC-0.8NS (the MFC dispersion was added with a weight ratio of 0.8 wt% non-spherical NPs) membranes, as shown in Figure 7. The porosity of the MFC-NS membrane remained similar to that of the pure MFC membrane, with a slight decrease for MFC-0.8NS, whereas the porosity of MFC-S membranes decreased slightly as the concentration of spherical silica with respect to MFC increased from 0.5 to 0.8 wt%. This could be attributed to tendency of spherical silica NPs to agglomerate at high concentrations, as indicated by the SEM analysis of the membranes.

**Figure 8:**
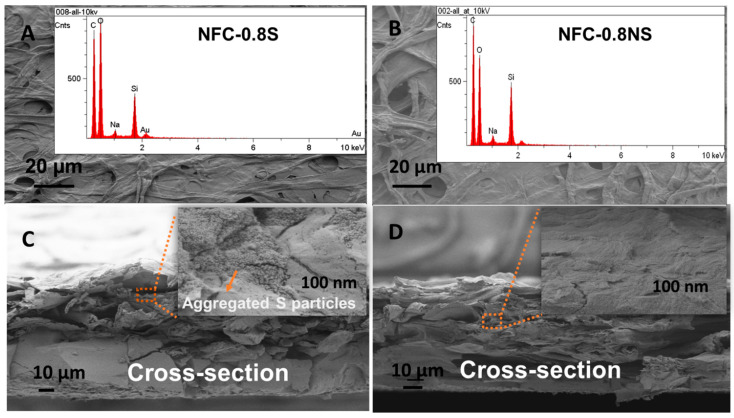
SEM images of (**A**) MFC-0.8S with EDS spectra (inset) and (**B**) MFC-0.8NS with EDS spectra (inset), and cross-section images of (**C**) MFC-0.8S and (**D**) MFC-0.8NS with magnified images (inset).

The morphology of the composite membranes was studied with SEM, where EDS mapping was used to detect the presence of added silica NPs in the membranes. Figure 8 shows the presence of silica peaks in the EDS spectra for both MFC-0.8NS and MFC-0.8S membranes. The cross-sectional analysis of the membranes done by SEM showed the aggregation of spherical silica NPs in the MFC-0.8S membrane (Figure 8C). However, there was no aggregation of non-spherical silica NPs detected in the cross-section images of MFC-0.8NS membranes (Figure 8D).

**Figure 9:**
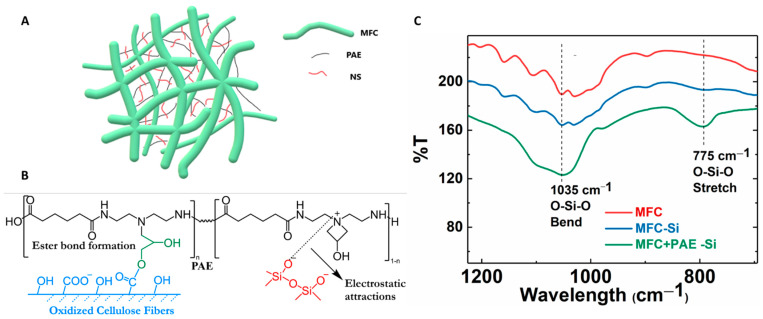
(**A**) The possible mechanism of silica NPs bridging between MFC nanofibers. (**B**) Illustration of the covalent bonding formed between the carboxylate group on MFC and the azetidinium group on PAE, as well as the electrostatic interaction between silica NPs and PAE. (**C**) FTIR analysis of MFC, MFC-NS (without PAE), and MFC-NS (with PAE) membranes.

In the composite membrane, the negatively charged silica NPs can be anchored by positively charged PAE molecules through electrostatic interaction [35]. In this case, silica NPs may act as physical cross-linkers between cationic nanofibrous network [22,36]. The possible cross-linking mechanism in MFC-NS membranes is illustrated in Figure 9A,B. The non-spherical silica NPs can form numerous cross-linking points with cationic PAE molecules, since NS NPs have abundant anionic sites and can enhance the integrality of the MFC scaffold. In contrast, spherical silica NPs offer less anionic sites for physical cross-linking, and thus, has a less enhancement effect than non-spherical silica NPs. This hypothesis can be verified by the FTIR results in Figure 9C. In this figure, it was found that the MFC-0.8NS membranes cross-linked with PAE exhibited the peak at 1035 cm^−1^, indicating the O-Si-O bending vibration, and at 775 cm^−1^, indicating the O-Si-O stretching vibration. In contrast, the IR spectra of MFC-0.8NS without the PAE addition and pure MFC did not show any O-Si-O peaks. These results confirmed the retention of anionic silica NPs by cationic PAE sites in the MFC scaffold.

The demonstrated composite cellulose membranes, containing silica NPs, exhibited superhydrophilicity in nature. It is known that the cellulose fibers are inherently hydrophilic, but the addition of hydrophilic anionic silica NPs also resulted in increasing the surface roughness, where the synergistic effect (surface roughness and hydrophilicity scaffold) leads to superhydrophilic the membrane. The comparison of the contact angle results and other membrane characteristics of MFC-S and MFC-NS membranes with those of commercial membranes is given in Table 3. It was seen that the contact angle of MFC-NS and MFC-S membranes was close to zero in the 20 s contact angle test, whereas the commercial PVDF (A6 and V6) and PES (PES LX) membranes exhibited much higher contact angles.

### 3.3. Wastewater Ultrafiltration and Antifouling Performance

**Figure 10:**
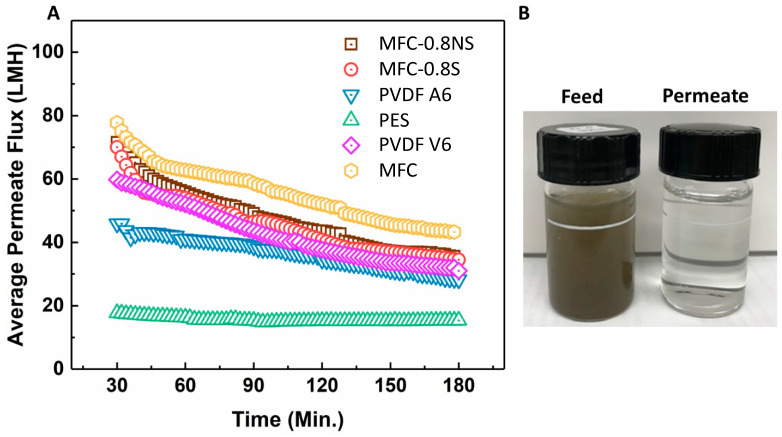
(**A**) The average permeate flux data for wastewater ultrafiltration at 7.5 psi pressure, (**B**) picture of feed wastewater and permeate after ultrafiltration using MFC-0.8NS membrane.

MFC, MFC-0.8NS, and MFC-0.8S membranes were tested with municipal wastewater and their UF performance was compared with that of commercial PVDF and PES membranes. The average permeate flux and separation efficiency results are shown in Figure 10. It was seen that the flux values for all MFC-based membranes as well as for commercial membranes all declined over time during ultrafiltration (3 h operation) due to the deposition of foulants on the membrane. The pure MFC membrane showed higher flux than MFC-0.8NS and MFC-0.8S membranes, which could be attributed to the higher porosity and larger pore size in the pure MFC membrane. Interestingly, MFC-0.8NS and MFC-0.8S membranes exhibited higher permeate flux than PVDF and PES membranes. The PES (PES-LX) membrane showed the lowest flux, which could be due to its lower porosity and smaller MWCO (molecular weight cut-off) pore size (Table 3).

**Figure 11:**
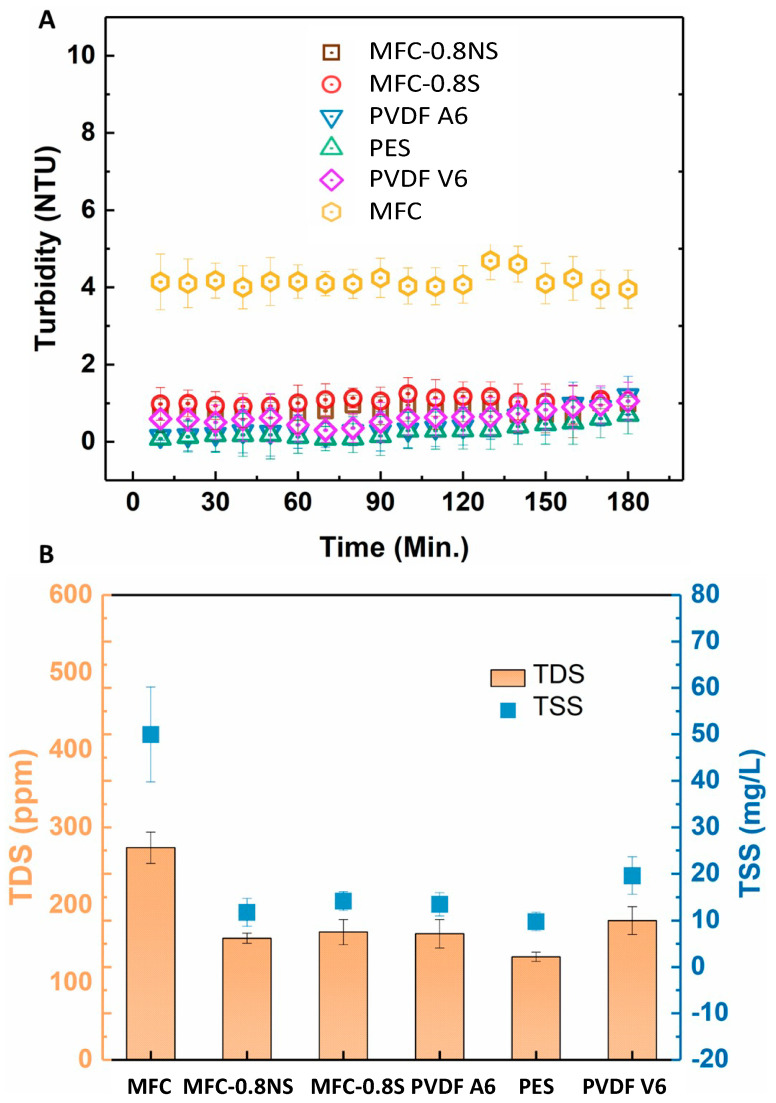
(**A**) Turbidity rejection and (**B**) total dissolved solids and total suspended solids rejection efficiency of MFC-based and commercial membranes.

The rejection efficiency of the membranes was measured in terms of turbidity, total dissolved solids (TDS), and total suspended solids (TSS). The rejection results are shown in the Figure 11. The pure MFC membrane showed lower rejection efficiency as compared to MFC-0.8NS and MFC-0.8S membranes due to larger effective pore size. The addition of silica nanoparticles to reduce the pore size effectively improved the rejection efficiency of the MFC-silica membranes (~99.9%) in terms of TSS and turbidity. These rejection results were similar to commercial PVDF and PES membranes.

**Figure 12:**
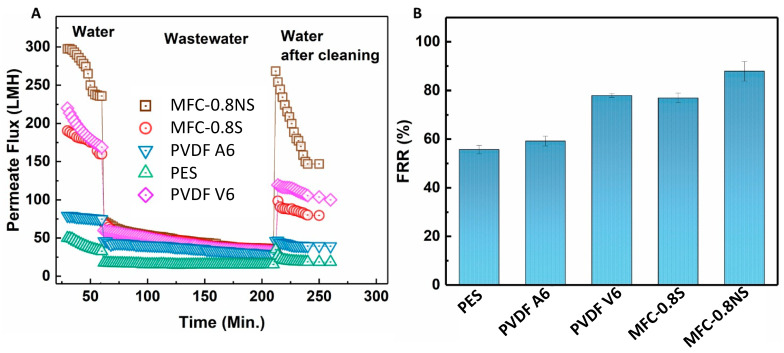
(**A**) Permeate flux for the fouling test and (**B**) flux recovery ratio (FRR) results for MFC-based and commercial membranes.

The antifouling capability of the MFC-silica NP membranes was evaluated against wastewater using the sequential ultrafiltration/washing method. The sequential UF results are shown in Figure 12A. The MFC-0.8NS showed highest water flux recovery post hydraulic cleaning of the membrane. However, the MFC-0.8S membrane exhibited slightly lower flux recovery after hydraulic cleaning step. The flux recovery ratio (FRR) of MFC-NS membrane was approximately 90%, while the MFC-S showed lower flux recovery ratio at approximately 77%, which was similar to PVDF V6 at 78% and higher than that of PVDF A6 and PES membranes (Figure 12B). The high flux recovery ratio means the foulant deposition could be easily removed from the membrane by cleaning process, and a lower value of FRR usually indicates irreversible fouling damage [8].

**Figure 13:**
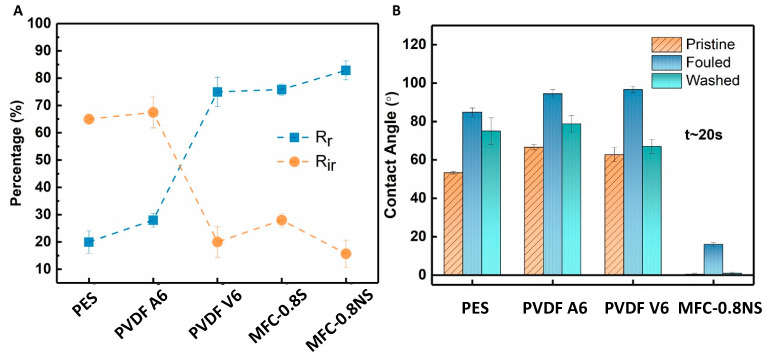
(**A**) Reversible and irreversible fouling ratios and (**B**) contact angle measurements of MFC and commercial membranes.

The total fouling of the membranes was determined in terms of reversible fouling ratio (R_r_) and irreversible fouling ratio (R_ir_) to examine the adherence of foulants to the membrane (Figure 13A). The MFC-NS membrane showed high reversible fouling ratio (~83%) and lower irreversible fouling (~15%). This could be attributed to the highly hydrophilic nature of the MFC-NS membrane, where simple hydraulic washing of the membrane was able to remove foulant deposition on the membrane. The PVDF A6 and PES membranes exhibited higher R_ir_ values (~65%) and lower R_r_ values (<30%), which implies that the fouling mechanism in the PVDF A6 and PES membranes was mostly irreversible and could not be recovered with membrane cleaning. However, the higher R_ir_ value for MFC-S than that for MFC-NS could be due to the agglomeration of spherical silica in the pores, which led to higher irreversible fouling due to pore-constriction. The membrane still showed higher reversible fouling, owing to its hydrophilic nature. This also indicates that non-spherical silica nanoparticles perform better than spherical particles in enhancing the antifouling property of the MFC membranes.

The contact angle measurements of pristine, fouled, and cleaned membranes were done to investigate the role of hydrophilicity of membranes in antifouling performance against wastewater (Figure 13B). The contact angle results showed the initial MFC-NS (also MFC-S) membrane was superhydrophilic (i.e., after 20 s, the contact angle was almost zero). In contrast, the contact angles for PVDF A6, PVDF V6, and PES were found to be 66.6°, 62.7°, and 53.3°, respectively, showing lower hydrophilicity in these commercial membranes. There was an increase in the contact angle of all the membranes after wastewater ultrafiltration due to the adherence of foulant particles to the membrane surfaces. However, the increase was observed to be more pronounced in the case of PVDF and PES membranes, making them even more hydrophobic. The higher fouling tendency of the PVDF and PES membranes could be due to hydrophobic nature of the scaffold with the hydrophobic species in the wastewater, which could not be fully recovered by the hydraulic cleaning of the membrane [17]. However, the hydraulic cleaning treatment of the fouled MFC-NS membranes was found to be effective, where the contact angle of the cleaned membrane was recovered back to almost zero. This indicates that the reversible fouling is the dominant fouling mechanism in the MFC-NS membrane. It is conceivable that the superhydrophilicity of the MFC-NS membrane can result in the formation of hydration layer on the membrane surface, which was responsible for the antifouling property [37,38].

### 3.4. Possible Fouling Mechanism of the MFC-NS Membrane

**Figure 14:**
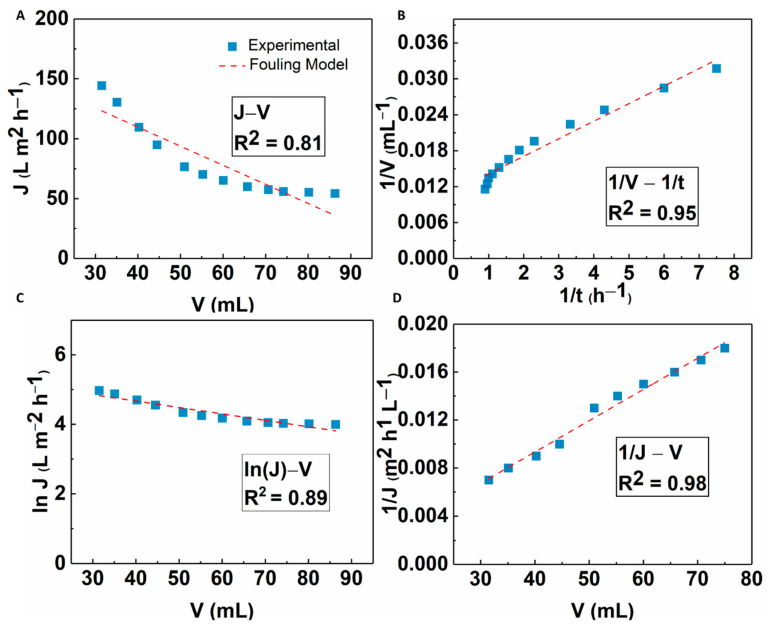
Fitting results of the flux decline analyzed by four fouling mechanism models: (**A**) complete blocking, (**B**) standard blocking, (**C**) intermediate blocking, and (**D**) cake filtration.

The fouling mechanism of the MFC-NS membrane was studied by classic filtration fouling models, as discussed earlier. The modeling results are shown in Figure 14. It was seen that cake filtration and standard blocking models all fitted well to the experimental results, with the cake filtration model showing the best fit results (R^2^ = 0.98). This implies that the fouling mechanism of the MFC-NS membrane during wastewater ultrafiltration is mainly due to the cake filtration, where both standard blocking and complete blocking mechanisms play a minor role. This is consistent with the recyclability study where the formation of cake layer due to sludge particles is responsible for the reversible fouling of the MFC-NS membrane, which can be easily removed by hydrolytic cleaning. The foulants in the wastewater sludge are complex, as they include suspended particles, macromolecules, and humic substances [8]. The modeling results further indicate that the fouling mechanism, although dominated by the cake layer formation, also involves pore blocking caused by adsorption of foulant particles, which leads to irreversible fouling.

### 3.5. Durability and Self-Healing Property of MFC-NS Membranes

**Figure 15:**
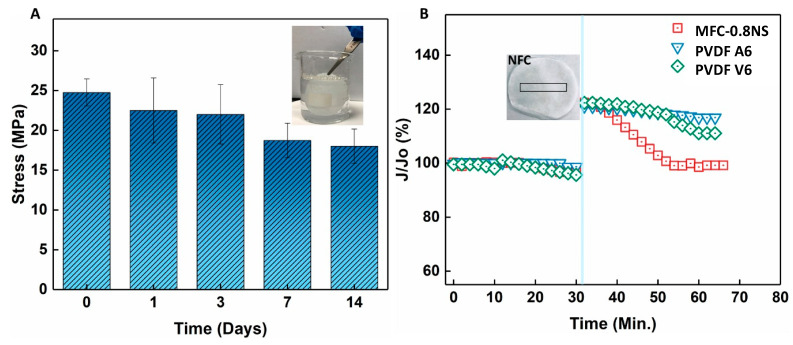
(**A**) Mechanical strength test of the MFC-0.8NS membrane after prolonged immersion in water and (**B**) self-healing property of the MFC-0.8NS membrane.

The mechanical strength and durability of the MFC-0.8NS membrane was investigated by immersing the membrane in water over a span of 14 days and the mechanical strength was evaluated at different time intervals using the dry membrane. Figure 15 shows that tensile stress of the MFC-0.8NS membrane decreased slightly around 7 and 14 days but maintained its mechanical stability, showing the durability of the MFC-NS membrane and the suitability for practical wastewater treatment. Figure 15B shows the self-healing property of the MFC-NS membrane. A small mechanical scratch was made on the MFC-NS and PVDF membranes after compacting the membranes with DI water and the scratched membrane was inserted in the dead-end cell for the filtration test. It was found that the MFC-NS membrane restored its flux over time, whereas the polymeric membranes failed to recover their initial flux. This could be attributed to the water absorption and swelling property of the MFC scaffold. This unique property of the cellulose fibers potentially makes them suitable membrane materials to handle minor wear and tear, pertaining to the harsh practical filtration environment.

## 4. Conclusions

In this study, we demonstrated the development of a highly hydrophilic and low-fouling membrane entirely derived from sustainable cellulose fibers with small additions of biocompatible additives in the form of PAE and silica nanoparticles. The MFC-silica membranes showed high hydrophilicity and high porosity. Both spherical and non-spherical silica nanoparticles were efficient to control pore size and improve rejection ratio. However, the non-spherical silica nanoparticles proved to be more suitable candidates, as their thread-like structure can form a higher content of physical cross-linkers with cationic PAE molecules, minimizing the nanoparticle aggregation and stabilizing the fibrous network structure. The MFC-NS membranes also exhibited high permeation flux compared to commercial PVDF and PES membranes as well as high separation efficiency (>99%) comparable to the commercial membranes. The hydrophilic surface of the cellulose membrane was effective in preventing fouling and exhibited a high flux recovery ratio (~90%). The investigation of the fouling mechanism in wastewater ultrafiltration using different fouling modeling suggested that cake layer formation is the major fouling mechanism in the MFC-NS membrane, where the pore blocking mechanism also plays a minor role in wastewater ultrafiltration.

## Figures and Tables

**Table 1:** Equations for different fouling mechanisms.

Fouling Model	Equation
**Complete Blocking**	$J_{O}-J=AV$
**Standard Blocking**	1t+B=JOV
**Intermediate Blocking**	$\ln J_{O}-\ln J=CV$
**Cake Filtration**	$\left(1/J\right)-\left(1/J_{O}\right)=DV$

Note: *J* is the permeate flux; *J_o_* is initial flux; *V* represents the filtrated volume; *t* represents the filtration time; A, B, C, and D are constants for different models.

**Table 2:** Apparent degree of delamination comparison at different homogenization conditions.

Fiber Conc. % (*w*/*w*) and No. of Passes	Pressure (bar)	Apparent Degree of Delamination, *c_N_* (%)	Estimated Energy Consumption (kWh/ton)
**0.6%, 2 Pass**	350	53.8 ± 2.1	3239
**0.5%, 1 Pass**	350	12.5 ± 4.4	1620
**0.9%, 1 Pass**	100	5.4 ± 2.5	310

**Table 3:** Comparison of MFC and commercial polymer membranes.

Membrane	Manufacturer	Porosity (%)	Thickness (µm)	Contact Angle (°)
**PES LX**	Sterlitech	49 ± 2.5	190	53.3 ± 1.1
**PVDF A6**	Sterlitech	50 ± 3.5	190	66.6 ± 1.5
**PVDF V6**	Sterlitech	65.2 ± 0.5	200	62.7 ± 3.8
**MFC-0.8NS**	-	58.5 ± 2.5	180	~0
**MFC-0.8S**	-	54.5 ± 1.3	150	~0

## Data Availability

Not applicable.
